# Impact of delisting high‐strength opioid formulations from a public drug benefit formulary on opioid utilization in Ontario, Canada

**DOI:** 10.1002/pds.4764

**Published:** 2019-03-14

**Authors:** Diana Martins, Wayne Khuu, Mina Tadrous, David N. Juurlink, Muhammad M. Mamdani, J. Michael Paterson, Tara Gomes, On behalf of the Ontario Drug Policy Research Network

**Affiliations:** ^1^ Li Ka Shing Knowledge Institute St. Michael's Hospital Toronto Ontario Canada; ^2^ ICES Toronto Ontario Canada; ^3^ The Sunnybrook Research Institute Toronto Ontario Canada; ^4^ Department of Medicine St. Michael's Hospital Toronto Ontario Canada; ^5^ Department of Medicine University of Toronto Toronto Ontario Canada; ^6^ Department of Pediatrics University of Toronto Toronto Ontario Canada; ^7^ Institute of Health Policy, Management, and Evaluation University of Toronto Toronto Ontario Canada; ^8^ Leslie Dan Faculty of Pharmacy University of Toronto Toronto Ontario Canada; ^9^ Department of Family Medicine McMaster University Hamilton Ontario Canada

**Keywords:** delisting, dose, evaluation, Ontario, opioids, pharmacoepidemiology, policy change

## Abstract

**Purpose:**

High‐strength opioid formulations were delisted (removed) from Ontario's public drug formulary in January 2017, except for palliative patients. We evaluated the impact of this policy on opioid utilization and dosing.

**Methods:**

We conducted a longitudinal study among patients receiving publicly funded, high‐strength opioids from August 2016 to July 2017. The primary outcome measure was weekly median daily opioid dose (in milligrams of morphine or equivalent; MME) of (1) publicly funded and (2) all opioid prescriptions irrespective of funding source, evaluated using interrupted time series analyses and stratified by palliative care status.

**Results:**

Following policy implementation, the weekly median daily dose of publicly funded opioids decreased immediately among non‐palliative patients by 10 MME (95% confidence limit [CL], −16.8 to −3.1) from a pre‐intervention dose of 424.5 MME (95% CL, 417.8‐431.2) and fell gradually among palliative patients by 3.9 MME per week (95% CL, −5.5 to −2.3) from a pre‐intervention dose of 450.1 MME (95% CL, 432.5‐467.7). In contrast, among all opioid prescriptions, gradual reductions in weekly median daily doses were observed only for non‐palliative patients, which decreased by 0.7 MME per week (95% CL, −1.3 to −0.2) from a pre‐intervention dose of 426.2 MME (95% CL, 420.9‐431.5).

**Conclusion:**

The delisting of publicly‐funded, high‐strength opioids was accompanied by changes in funding source and small reductions in the weekly median daily doses dispensed. Although observed dose reductions of less than 1 MME weekly are likely not clinically relevant, safety implications of these changes require further monitoring.

**KEY POINTS**

- Among non–palliative care patients, delisting publicly funded high‐strength opioids led to an immediate reduction in the weekly median daily dose of publicly funded opioids dispensed and an accelerated declining dose of all opioids dispensed (irrespective of payer).
- Among palliative care patients, there were no significant changes in weekly median daily dose following the policy when considering all opioid prescriptions dispensed.
- One in three non–palliative care patients and one in five palliative care patients transitioned to accessing high‐strength opioids through private insurance or by paying out of pocket.
- Overall, we found no evidence of the policy leading to increased likelihood of complete opioid discontinuation in any patient population.

## 1 INTRODUCTION

High‐strength opioid formulations are often used to treat severe pain when lower doses fail to produce adequate analgesia. Yet higher doses of opioids are associated with numerous harms, including motor vehicle collisions,^1^ opioid misuse,^2^, ^3^ hyperalgesia,^4^ depression,^5^ testosterone suppression,^6^ suicide,^7^ and nonfatal or fatal overdose.^8^, ^9^, ^10^, ^11^, ^12^ Given these concerns, the current guidelines for opioid use for chronic noncancer pain in Canada^13^ and the United States^11^ recommend against escalating beyond 90 mg of morphine or equivalent (MME) per day and suggest carefully tapering high doses when harms outweigh benefits. To regulate the availability of high‐strength opioids in communities and help reduce high opioid doses, some jurisdictions in Canada and the United States have restricted access to high‐strength opioid formulations^14^ or prescriptions with high daily doses.^15^ A 2017 petition to the US Food and Drug Administration (FDA) called for banning opioid formulations that could achieve daily doses of 90 MME or more when taken as directed.^16^

In Ontario, Canada, 40% of long‐acting opioid prescriptions dispensed in 2016, a period immediately preceding the release of the new Canadian guidelines, were for daily doses exceeding 90 MME,^17^ and approximately 4% of newly treated patients were initiated on a dose exceeding this threshold.^18^ On January 31, 2017, Ontario's Public Drug Program (OPDP) delisted all high‐strength opioid formulations that were listed on their formulary at the time, which included 75‐ and 100‐mcg/h fentanyl patches, 24‐ and 30‐mg hydromorphone capsules, and 200‐mg morphine tablets. By delisting these products, they were no longer eligible for reimbursement from the public drug program. This change was implemented as part of a strategy to reduce the risk of addiction and opioid‐related adverse events resulting from the misuse and diversion of these opioids.^14^ An exception was made for palliative care patients^19^ who could access these formulations through a prior authorization process or from physicians registered with the palliative care facilitated access (PCFA) program. The new policy did not impact coverage for lower strength opioid formulations that could be combined to achieve equivalent daily doses. In addition, high‐strength opioids could still be obtained outside the public drug plan (ie, out of pocket and private insurers).

We conducted an evaluation to assess whether this delisting policy led to any potentially harmful changes in opioid dispensing patterns. Specifically, we sought to evaluate the impact of this policy on opioid prescription utilization and dosing among public drug beneficiaries in Ontario who were already taking these medications.

## 2 METHODS

### 2.1 Setting and design

We conducted a population‐based, longitudinal study among a prevalent cohort of Ontarians dispensed publicly funded, high‐strength opioids between August 1, 2016, and July 31, 2017. This permitted a 6‐month prepolicy accrual period and a 6‐month postpolicy observation period. In Ontario, individuals are eligible for publicly funded medications if they are aged 65 years and older, have high drug costs relative to their income, are unemployed, receive disability support or home care, or live in a long‐term care facility.

### 2.2 Data sources

We used the Ontario Narcotic Monitoring System database to identify all opioid prescriptions dispensed from pharmacies in Ontario and the Ontario Drug Benefit (ODB) claims database to categorize prescriptions that were publicly funded. We used the Ontario Health Insurance Plan (OHIP) Claims History Database and OHIP Registered Persons Database to examine demographic characteristics and vital status. We identified emergency department (ED) visits and hospitalizations using the Canadian Institute for Health Information (CIHI) National Ambulatory Care Reporting System and CIHI Discharge Abstract Database, respectively. We used the OHIP Claims Database to capture all physician office visits and to identify individuals receiving palliative care services. All records were linked using unique, encoded identifiers and analyzed at ICES (www.ices.on.ca). Use of these data was authorized under section 45 of Ontario's Personal Health Information Protection Act, which does not require review by a research ethics board.

### 2.3 Study population

We constructed a cohort comprising all individuals affected by the policy implementation, defined as those who received a publicly funded prescription for a high‐strength opioid where 120% of the days' supply overlapped the policy implementation date (January 31, 2017). This 20% grace period for the days' supply was used to account for late refills and incomplete adherence given the PRN (ie, “use as needed”) nature of some opioids. To limit the cohort to chronic high‐strength opioid recipients, we excluded individuals who were not receiving a prescription high‐strength opioid at the start of the study period (6 months prior to policy implementation). We excluded individuals who died before the end of the follow‐up period to prevent observing changes in dose that could be due to having a person's dose included in the prepolicy period and not in the postpolicy period. This is necessary as we do not allow new patients in the follow‐up period due to our focus on those affected by the policy at the time of implementation. Cohort entry was defined as the date of the last high‐strength opioid prescription dispensed prior to the policy implementation date. We stratified the cohorts by palliative care status, defined using physician billing codes for palliative care services (Appendix S1) in the 6 months prior to cohort entry. To test the specificity of our findings, we used the same methods to construct a historical cohort 1 year earlier (August 1, 2015, and July 31, 2016), using January 31, 2016, as the “dummy” policy implementation date.

### 2.4 Patient characteristics

We reported baseline patient characteristics for each cohort, stratified by palliative care status, including age, sex, residence in a rural community or long‐term care home, and neighborhood income quintile. We also report eligibility for the public drug program, categorized into seniors, long‐term care residents, and individuals enrolled in disability and other social assistance programs (ie, high drug costs relative to income, employment assistance, home care, resident of home for special care, or enrolled in the Ontario Disability Support Program). Furthermore, we measured health service utilization in the 6 months prior to cohort entry, including hospitalizations, ED visits, and physician visits.

### 2.5 Outcomes

Outcomes were assessed by including all opioid prescriptions dispensed to individuals in the cohort over the study period. In the primary analysis, we assessed changes in weekly median daily opioid dose (in MME) during the study period. Specifically, we calculated the average daily opioid dose for each person every week, defined as the sum of the daily dose for the days covered by the prescription divided by the number of days covered by the prescription in the same week. We reported the median of this measure across patients dispensed opioids each week, resulting in one group‐level summary estimate per week that we refer to as weekly median daily dose. This analysis was conducted for (1) publicly funded prescriptions and (2) all prescriptions dispensed, irrespective of funding source.

In a series of secondary analyses, we assessed changes in opioid utilization using prescriptions dispensed at any point in the 6 months following the policy. These binary outcomes included the following: continued use of publicly funded high‐strength opioids, continued use of high‐strength opioids paid through other means, discontinuation (ie, no prescription) of any publicly funded opioids, discontinuation of any opioids paid through any means, and de novo initiation of buprenorphine/naloxone or methadone.

### 2.6 Statistical analysis

For our primary outcome, we used interrupted time series analyses and fit linear segmented regression models to the population weekly measure of median opioid daily dose, which are commonly used to assess the impact of policies/interventions on time series trends.^20^, ^21^, ^22^, ^23^, ^24^ We included parameters to estimate the pre‐intervention dose (intercept), pre‐intervention trend (slope), post‐intervention change in level (step), and post‐intervention change in trend. In the presence of autocorrelation, we used a backward stepwise approach to include autoregressive parameters in the model. For our secondary binary outcomes, we used logistic regression models to test for differences between the intervention and historical cohorts. For this secondary analysis, we used generalized estimating equations to account for the non‐independence of observations since some individuals were represented in both cohorts. We stratified all analyses by palliative care status and used a type 1 error rate of 0.05 as the threshold for statistical significance. All analyses were conducted using SAS software (version 9.4; SAS Institute Inc, Cary, North Carolina).

## 3 RESULTS

We included 3763 individuals in the intervention cohort and 6892 individuals in the historical cohort following exclusions (Appendix S2), among whom few were receiving palliative care (2.9% [N = 109] and 2.1% [N = 143], respectively). Baseline characteristics and health service utilization measures were similar between individuals in the intervention and historical cohorts among both palliative care and non‐palliative care (Table 1).

**Table 1:** Baseline characteristics of publicly funded high‐strength opioid recipients, stratified by study cohort and palliative care status^a^

	Palliative care		Non‐palliative care	
	Historical cohort	Intervention cohort	Historical cohort	Intervention cohort
Baseline characteristics	N = 143	N = 109	N = 6749	N = 3654
Age, N (%)				
0‐24	0 (0%)	0 (0%)	14 (0.2%)	≤5 (≤0.1%)
25‐44	16 (11.2%)	≤5 (≤4.6%)	869 (12.9%)	403‐407 (11‐12%)
45‐64	57 (39.9%)	46‐50 (42‐46%)	3159 (46.8%)	1713 (46.9%)
65+	70 (49.0%)	58 (53.2%)	2707 (40.1%)	1533 (42.0%)
Male, N (%)	65 (45.5%)	53 (48.6%)	3058 (45.3%)	1714 (46.9%)
Rural residence, N (%)	24 (16.8%)	12 (11.0%)	1174 (17.4%)	709 (19.4%)
Neighborhood income quintile (N, %)				
Q1 (lowest)	23 (16.1%)	24 (22.0%)	2131 (31.6%)	1090 (29.8%)
Q2	43 (30.1%)	22 (20.2%)	1510 (22.4%)	847 (23.2%)
Q3	34 (23.8%)	28 (25.7%)	1224 (18.1%)	686 (18.8%)
Q4	26 (18.2%)	16 (14.7%)	1021 (15.1%)	560 (15.3%)
Q5 (highest)	17 (11.9%)	19 (17.4%)	820 (12.1%)	455 (12.5%)
LTC resident	6 (4.2%)	0 (0%)	531 (7.9%)	109 (3.0%)
Public drug program eligibility^b^				
1. Disability and other social assistance programs	85 (59.4%)	69 (63.3%)	4179 (61.9%)	1212 (60.5%)
2. Seniors	52 (36.4%)	40 (36.7%)	2206 (32.7%)	1360 (37.2%)
3. Long‐term care residence (nursing homes)	6 (4.2%)	0 (0.0%)	364 (5.4%)	82 (2.2%)
Physician office visits in past 6 mo, N (%)				
1+	140 (97.9%)	107 (98.2%)	6134 (90.9%)	3390 (92.8%)
0‐4	27 (18.9%)	16 (14.7%)	3205 (47.5%)	1668 (45.6%)
5‐10	48 (33.6%)	38 (34.9%)	2322 (34.4%)	1334 (36.5%)
11+	68 (47.6%)	55 (50.5%)	1222 (18.1%)	652 (17.8%)
Hospitalization in past 6 mo, N (%)	51 (35.7%)	35 (32.1%)	767 (11.4%)	398 (10.9%)
Emergency department visit in past 6 mo, N (%)	73 (51.0%)	56 (51.4%)	2071 (30.7%)	1076 (29.4%)

a In cases where the number of users is less than six, this number has been suppressed to ensure confidentiality. In cases where there is only one record being suppressed, another record has been suppressed to provide a range in order to avoid residual disclosure.

b Public drug program eligibility assigns individuals hierarchically as follows: receiving high drug costs relative to income, high‐income seniors, resident of home for special care, receiving home care, resident of long‐term care, receiving employment assistance, enrolled in the Ontario Disability Support Program, and low‐income seniors. For this reason, the numbers may differ slightly from those identified as seniors and those living in a long‐term care residence.

### 3.1 Daily opioid dose

#### 3.1.1 Patients not receiving palliative care

**Figure 1:**
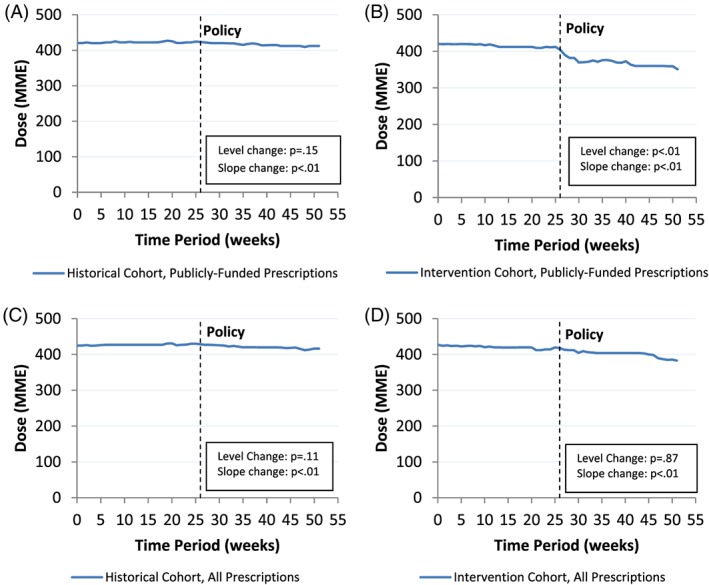
Time trends in the median daily opioid dose before and after the policy implementation among non–palliative care patients. A, Publicly funded prescriptions in the historical cohort; B, publicly funded prescriptions in the intervention cohort; C, all prescriptions in the historical cohort; D, all prescriptions in the intervention cohort [Colour figure can be viewed at wileyonlinelibrary.com]

Prior to the policy, patients who were not receiving palliative care were prescribed a weekly median daily opioid dose of 424.5 MME (95% confidence limit [CL], 417.8‐431.2) from publicly funded prescriptions and 426.2 MME (95% CL, 420.9‐431.5) when considering all prescriptions dispensed (Table 2 and Figure 1). Introduction of the policy was associated with an immediate 10.0 MME reduction in the weekly median daily dose of publicly funded opioids (95% CL, −16.8 to −3.1) and an additional reduction of 0.9 MME per week (95% CL, −1.6 to −0.3) in the 6 months following the policy. In contrast, there was no immediate reduction in weekly median daily dose of all opioids dispensed (*P* = 0.87). Yet, among all opioid prescriptions dispensed, we observed a small, but statistically significant reduction in weekly median daily dose following policy implementation (−0.7 MME per week; 95% CL, −1.3 to −0.2). In the historical cohort, the dummy policy date was associated with a similar, significant reduction in the weekly median daily opioid dose of 0.6 MME per week (95% CL, −0.7 to −0.5) for publicly funded opioid prescriptions and 0.7 MME per week (95% CL, −0.8 to −0.6) among all opioid prescriptions dispensed.

**Table 2:** Segmented regression model for weekly median daily opioid doses (MME) per patient in the historical and intervention cohorts, among non–palliative care patients^a^

Opioid source	Variable (in MME)	Historical cohort (N = 6749)		Intervention cohort (N = 3654)	
		Estimate (95% CL)	*P* Value	Estimate (95% CL)	*P* Value
Publicly‐funded opioids	Baseline dose	420.9 (419.7‐422.1)	–	424.5 (417.8‐431.2)	–
	Pre‐intervention dose trend	0.1 (0.03‐0.2)	–	−0.8 (−1.2 to −0.4)	–
	Intervention level change in dose	−1.2 (−2.8 to 0.5)	0.15	−10.0 (−16.8 to −3.1)	<0.01
	Intervention trend change in dose	−0.6 (−0.7 to −0.5)	<0.01	−0.9 (−1.6 to −0.3)	<0.01
All opioids	Baseline dose	425.1 (424.0‐426.2)	–	426.2 (420.9‐431.5)	–
	Pre‐intervention dose trend	0.2 (0.07‐0.2)	–	−0.4 (−0.7 to −0.07)	–
	Intervention level change in dose	−1.3 (−2.8 to 0.3)	0.11	0.4 (−4.5 to 5.3)	0.87
	Intervention trend change in dose	−0.7 (−0.8 to −0.6)	<0.01	−0.7 (−1.3 to −0.2)	<0.01

a A *P* value < 0.05 indicates a statistically significant change in dose level or trend.

#### 3.1.2 Patients receiving palliative care

**Figure 2:**
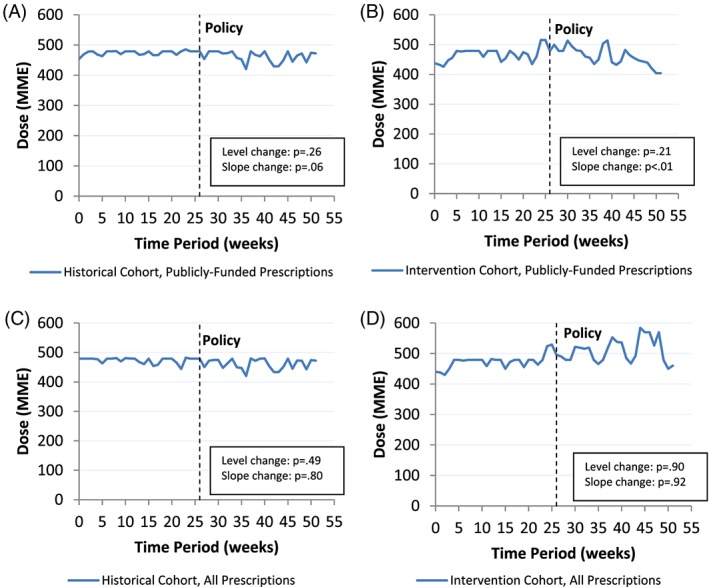
Time trends in the median daily opioid dose before and after the policy implementation among palliative care patients. A, Publicly funded prescriptions in the historical cohort; B, publicly funded prescriptions in the intervention cohort; C, all prescriptions in the historical cohort; D, all prescriptions in the intervention cohort [Colour figure can be viewed at wileyonlinelibrary.com]

Prior to the policy, patients receiving palliative care in the intervention cohort received a weekly median daily opioid dose of 450.1 MME (95% CL, 432.5‐467.7) from publicly funded prescriptions and 454.1 MME (95% CL, 433.6‐474.5) when considering all prescriptions dispensed (Table 3 and Figure 2). Following the policy, the weekly median daily dose of publicly funded opioids began to decrease (compared with an increasing trend prepolicy), reflecting a slope change of −3.9 MME per week (95% CL, −5.5 to −2.3). When considering all opioid prescriptions regardless of payer, there were no significant changes in weekly median daily dose following the policy. In the historical cohort, the dummy policy date was not associated with any significant changes in weekly median daily dose of opioids dispensed.

**Table 3:** Segmented regression model for weekly median daily opioid doses (MME) per patient in the historical and intervention cohorts, among palliative care patients^a^

Opioid source	Variable (in MME)	Historical cohort (N = 143)		Intervention cohort (N = 109)	
		Estimate (95% CL)	*P* Value	Estimate (95% CL)	*P* Value
Publicly funded opioids	Baseline dose	469.4 (458.6‐480.2)	–	450.1 (432.5‐467.7)	–
	Pre‐intervention dose trend	0.4 (−0.3 to 1.06)	–	1.2 (0.09‐2.4)	–
	Intervention level change in dose	−8.4 (−23.2 to 6.5)	0.26	15.5 (−9.0 to 40.03)	0.21
	Intervention trend change in dose	−1.0 (−2.0 to 0.02)	0.06	−3.9 (−5.5 to −2.3)	<0.01
All opioids	Baseline dose	477.8 (466.2‐489.5)	–	454.1 (433.6‐474.5)	–
	Pre‐intervention dose trend	−0.3 (−1.08 to 0.4)	–	1.5 (0.1‐2.8)	–
	Intervention level change in dose	−5.6 (−21.7 to 10.5)	0.49	1.7 (−26.7 to 30.09)	0.90
	Intervention trend change in dose	0.1 (−0.9 to 1.2)	0.80	−0.1 (−2.0 to 1.8)	0.92

a A *P* value < 0.05 indicates a statistically significant change in dose level or trend.

### 3.2 Patterns of opioid utilization

#### 3.2.1 Patients not receiving palliative care

Among non–palliative care patients in the intervention cohort, 33.2% (n = 1212) transitioned to accessing high‐strength opioids through non–publicly funded means (compared with 0.2% in the historical cohort, *P* < 0.01; Table 4). Despite this, the prevalence of discontinuation of any publicly funded opioid was low, but significantly higher in the intervention cohort (5.3%) compared with the historical cohort (0.5%; *P* < 0.01). We observed no difference between the intervention and historical cohorts in overall rates of opioid discontinuation among all opioids (0.6% vs 0.4%; *P* = 0.17) or incidence of methadone or buprenorphine/naloxone initiation after the policy (1.6% vs 1.4%; *P* = 0.35).

**Table 4:** Impact of the policy on access to opioids, stratified by study cohort and palliative care status^a^

	Palliative care			Non‐palliative care		
	Historical cohort	Intervention cohort		Historical cohort	Intervention cohort	
Measured in the 6 mo postpolicy period	N = 143	N = 109	*P* Value	N = 6749	N = 3654	*P* Value
Receipt of a publicly funded high‐strength opioid	141 (98.6%)	54 (49.5%)	<0.01	6624 (98.1%)	85 (2.3%)	<0.01
Receipt of a high‐strength opioid from non–publicly funded source only	≤5 (≤3.5%)	23 (21.1%)	<0.01	14 (0.2%)	1212 (33.2%)	<0.01
No publicly funded opioid prescription	≤5 (≤3.5%)	≤5 (≤4.5%)	0.23	36 (0.5%)	194 (5.3%)	<0.01
No opioid prescription from any payer	0 (0%)	0 (0%)	‐	26 (0.4%)	21 (0.6%)	0.17
Initiation of methadone or buprenorphine/naloxone from any payer	≤5 (≤3.5%)	≤5 (≤4.5%)	0.85	95 (1.4%)	59 (1.6%)	0.35

a A *P* value < 0.05 indicates a statistically significant difference between groups measured. In cases where the number of users is less than six, this number has been suppressed to ensure confidentiality.

#### 3.2.2 Patients receiving palliative care

Approximately half (49.5%; n = 54) of palliative care patients in the intervention cohort continued to access publicly funded high‐strength opioids following the policy, which was significantly lower than in the preceding year (98.6%; *P* < 0.01; Table 4). In contrast, a much higher percentage (21.1%; n = 23) transitioned to accessing these opioids through other means relative to the preceding year (less than or equal to 3.5%; *P* < .01).

## 4 INTERPRETATION

Following Ontario's delisting of high‐strength opioid formulations from its public drug program, we found statistically significant reductions in publicly funded weekly median daily opioid doses, which were more substantial among patients not receiving palliative care. However, when considering opioids reimbursed through any means, reductions in weekly median daily dose were very small (change of less than 1 MME reduction per week), statistically significant only among patients not receiving palliative care, and present in both the intervention and historical cohorts. Although we observed changing patterns of access for high‐strength opioids through public and private payers, we found no evidence of complete opioid discontinuation following the policy. These findings are similar to an Oregon study evaluating the impact of a prior authorization policy for opioid prescriptions exceeding 120 MME per day, which reported a 20.3% decline in the probability of receiving a high‐dose opioid prescription.^15^ However, in contrast to our study, the study by Hartung et al did not examine the impact on dose received and was unable to measure the extent to which prescriptions were paid out of pocket.

A key finding of our study is that, although the majority of non–palliative care patients impacted by the policy transitioned to similar opioid doses using lower strength formulations paid for by the public drug plan, one‐third transitioned to accessing high‐strength opioid formulations outside of the public drug plan. While we are unable to determine factors associated with this decision, it may be reflective of individuals with private insurance who preferred to take high‐strength formulations, or could be representative of individuals who continued to access these formulations for the purpose of diversion. Additionally, the policy led to a small but significantly accelerated rate of decrease in overall daily opioid doses dispensed, which suggests that clinicians may have used the delisting, alone or together with other programs and guidelines, as an opportunity to safely taper opioid doses. Given this finding, it is reassuring that weekly reductions in dose remained small, suggesting no evidence of widespread rapid tapering associated with the introduction of this policy. Interestingly, we observed a similar trend in the historical cohort, which may have be influenced by the CDC guidelines^11^ released in the month following our dummy policy date.

The finding that one in five palliative care patients transitioned to accessing high‐strength opioids from nonpublic sources following the policy may suggest that some physicians were unaware of the policy's exception for these patients, or of the mechanisms to request access. However, because daily opioid doses did not fall appreciably when considering all prescriptions irrespective of payer, it appears the policy did not adversely impact the care of palliative care patients. Despite this, future research should investigate the longer term patterns of high‐strength opioid use in this population to confirm whether these findings are temporary or representative of a permanent shift in funding for high‐strength opioids in palliative care.

### 4.1 Strengths and limitations

Our ability to identify all opioids dispensed in Ontario allows us to evaluate the impact of a delisting policy at a population level. However, some limitations warrant discussion. First, analyses were limited to individuals with a valid Ontario health card (97% of NMS prescriptions), which could lead to a small degree of misclassification of continued opioid use. Second, our definition of palliative care, while broad, might not have captured all such patients in Ontario. This is evidenced by our finding that 2.3% of individuals identified as being non–palliative care patients continued to receive publicly funded, high‐strength opioids following the policy. It is possible that these patients were in fact palliative care patients, or that they were treated by a PCFA physician who wrote a prescription for these medications inappropriately. In both of these cases, the null findings in our historical cohort analyses suggest that these limitations were not likely to have influenced our main findings.

## 5 CONCLUSION

Delisting publicly funded, high‐strength opioids in Ontario, Canada, led to changes in patterns of opioid access and small reductions in dose among individuals receiving these medications, which were largely concentrated among non–palliative care patients. Future work should examine whether changes in utilization among palliative care patients are transitional as prescribers adapt to the policy, as well as whether the policy had any safety implications or impact on the prevalence of opioid recipients reaching high daily opioid doses.

## ETHICS STATEMENT

Use of these data was authorized under section 45 of Ontario's Personal Health Information Protection Act, which does not require review by a Research Ethics Board.

## CONFLICT OF INTEREST

Muhammad Mamdani has received honoraria from NovoNordisk, Allergan, and Celgene. David Juurlink is a volunteer member of Physicians for Responsible Opioid Prescribing and has received payment for expert testimony related to opioids. Tara Gomes, Mina Tadrous, David Juurlink, and Muhammad Mamdani have received grant funding from the Ontario Ministry of Health and Long‐Term Care. All other authors report no conflicts of interest.

## Supporting information

Data S1
Appendix S1. OHIP physician billing codes used to define palliative care
Appendix S2: Exclusions used to create cohorts
Appendix S3: Average number of weeks receiving an opioid prescription per person
