# Supplementary material for: Impact of delisting high‐strength opioid formulations from a public drug benefit formulary on opioid utilization in Ontario, Canada
Source: Pharmacoepidemiol Drug Saf. 2019 Mar 14;28(5):726–33. doi: 10.1002/pds.4764 (PMC6518867; doi:10.1002/pds.4764)
Supplement: Supplementary file 1 — Data S1 Appendix S1. OHIP physician billing codes used to define palliative care Appendix S2: Exclusions used to create cohorts Appendix S3: Average number of weeks receiving an opioid prescription per person [file PDS-28-726-s001.docx]

**Appendix 1. OHIP physician billing codes used to define palliative care**

| **OHIP Fee Code** | **Description** |
| --- | --- |
| A945 | GEN./FAM.PRACT.SPECIAL PALLIATIVE CARE CONSULTATION |
| B998 | SPEC VIS PALLIATIVE CARE HOME, DAYS, EVE |
| C945 | SPECIAL PALLIATIVE CARE CONSULT HOSP IN PATIENT |
| C882 | TERMINAL CARE IN HOSP.G.P/F.P |
| C982 | PALLIATIVE CARE |
| K023 | PALLIAT CARE SUPPORT INDIVID CARE 1/2 HR OR MAJOR PART |
| W872 | TERMINAL CARE N.H G.P/FAMILY PRACTICE |
| W882 | TERMINAL CARE IN CHR.HOSP.G.P. |
| W972 | PALLIATIVE CARE |
| W982 | PALLIATIVE CARE |

**Appendix 2: Exclusions used to create cohorts**

| **Exclusions** | **Palliative Care** | | **Non-Palliative Care** | |
| --- | --- | --- | --- | --- |
|  | **Intervention Cohort** | **Historical Cohort** | **Intervention Cohort** | **Historical Cohort** |
| Eligible for refill in post-policy period | **N=252** | **N=364** | **N=4153** | **N=7977** |
| Died prior to end of study period | 90 (35.7%) | 122 (33.5%) | 118 (2.8%) | 269 (3.4%) |
| No high-strength opioid at start of study | 53 (21.0%) | 99 (27.2%) | 381 (9.2%) | 959 (12.0%) |
| **Included in study:** | **109 (43.3%)** | **143 (39.3%)** | **3,654 (88%)** | **6,749 (84.6%)** |

**Appendix 3: Average number of weeks receiving an opioid prescription per person**

| **Number of weeks receiving an opioid prescription in the study period (mean (standard deviation))** | **Palliative Care** | | **Non-Palliative Care** | |
| --- | --- | --- | --- | --- |
|  | **Intervention Cohort** | **Historical Cohort** | **Intervention Cohort** | **Historical Cohort** |
| **Publicly-funded opioids** | 28.2 (9.9) | 28.5 (9.6) | 34.5 (12.4) | 34.5 (12.7) |
| **All Opioids** | 28.6 (9.8) | 29.1 (9.4) | 35.0 (12.5) | 35.3 (12.6) |
